# RemScan: A tool for monitoring the bioremediation of Total Petroleum Hydrocarbons in contaminated soil

**DOI:** 10.1016/j.mex.2018.06.019

**Published:** 2018-07-03

**Authors:** Leadin S. Khudur, Andrew S. Ball

**Affiliations:** Centre for Environmental Sustainability and Remediation, School of Science, RMIT University, Bundoora, VIC, 3083, Australia

**Keywords:** RemScan for monitoring bioremediation, Soil contamination, Accurate TPH assessment, Cost-effective technique

## Abstract

Total Petroleum Hydrocarbons (TPH) represent major environmental contaminants which pose a significant risk to ecosystems and humans heath if left untreated. Bioremediation represents a simple, cheap and environmentally-safe approach to clean up TPH-contaminated sites. Traditional TPH analysis is expensive and time-consuming. Here we assess, for the first time, the potential of RemScan as a fast, accurate and cost-effective portable device to be used as a tool to monitor the bioremediation process. A variety of TPH-contaminated soils were subject to TPH quantitative analysis using RemScan. The TPH values obtained were validated and compared against the results obtained from an accredited external laboratory, which uses Gas Chromatography / Mass Spectrometry (GC/MS) for TPH analysis.

• RemScan showed a correlation coefficient (R^2^) of 0.998 in comparison with the traditional methods, but importantly with a significant reduction in both time and cost.
• RemScan was successfully used to measure TPH concentrations in bioremediated, weathered-contaminated and highly contaminated soil samples with TPH concentrations varying from 100 to 100,000 mg kg^−1^.
• The RemScan Laboratory Station was used to minimize the source of errors associated with human manual handling.

## Graphical abstract

**Figure:**
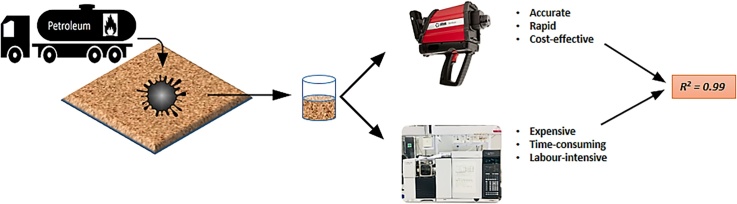

**Specifications Table**

**Table:** 

Subject area	*Environmental Science*
More specific subject area	*Bioremediation of hydrocarbon-contaminated soil*
Method name	*RemScan for monitoring bioremediation*
Name and reference of the original method	*WEBSTER, G. T., SORIANO-DISLA, J. M., KIRK, J., JANIK, L. J., FORRESTER, S. T., MCLAUGHLIN, M. J. & STEWART, R. J. 2016. Rapid prediction of total petroleum hydrocarbons in soil using a hand-held mid-infrared field instrument. Talanta, 160, 410-416.*

## Method background

This study aimed to validate the RemScan to be used as an accurate, cost-effective and prompt tool for monitoring the bioremediation of TPH-contaminated soil. To the best of the authors’ knowledge, the performance of RemScan in determining TPH concentration during a bioremediation process compared with traditional laboratory analysis has never been reported. The significance of this study is therefore that it represents the first to validate the use of the RemScan to evaluate the efficacy of the bioremediation of a TPH contaminated soil. In this study, a variety of bioremediation treatments were set up and soil samples were collected at different times over 150 days for quantitative analysis of TPH concentrations by an accredited laboratory using conventional GC/MS. In addition, we used the RemScan device to determine the concentration of TPH over a broad concentration range (100 - 100,000 mg kg^−1^).

## Method details

**Fig. 1:**
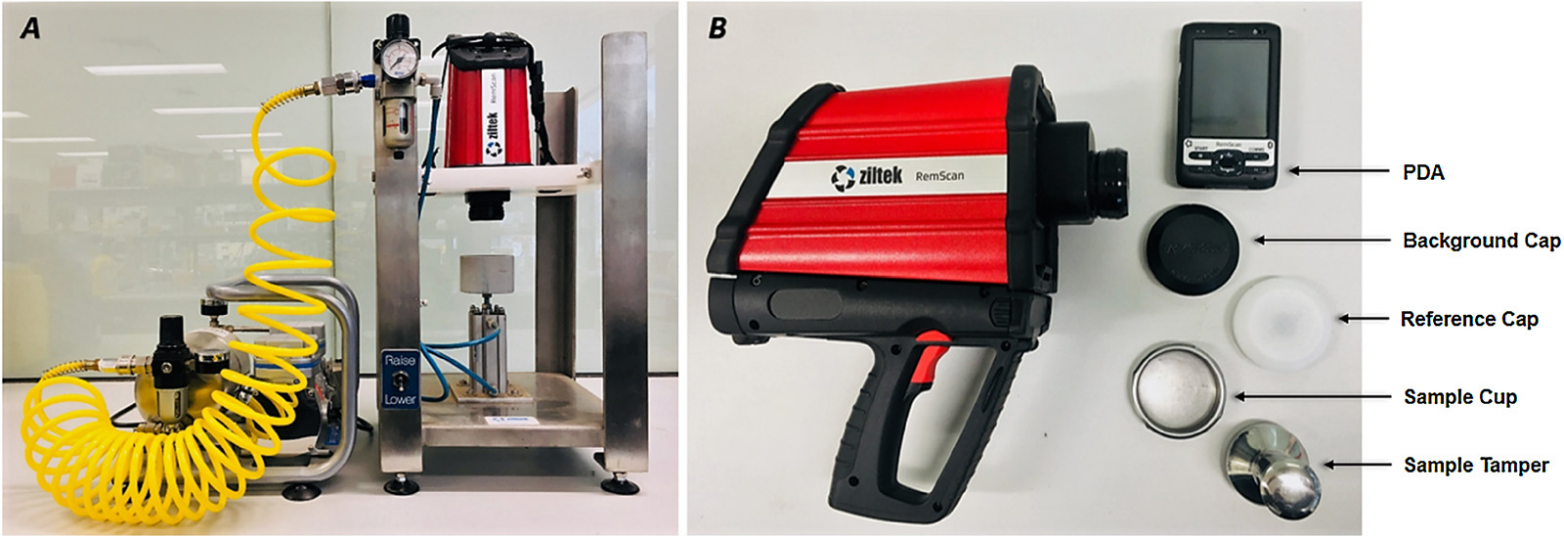
(A) RemScan device installed on the Lab Station. (B) RemScan device and the related components.
11 The device was connected to the Personal Digital Assistant (PDA) (Fig. 1.B) by pressing the “START” button on the PDA device.
12 After waiting around 15 min for the RemScan to warm up, the time and date were corrected on the PDA device using the digital pen provided.
13 The “Background cap” was placed on the nose-corn and scanned for about 1 min as instructed on the PDA. Similarly, the “Reference cap” was scanned and then the device was ready to scan the soil samples.
14 *Scanning the soil samples*
15 30 – 50 g of the already prepared soil sample was placed in the “Sample Cup” provided, mixed thoroughly using a spatula and then tamped using the “Sample tamper” provided until a completely flat surface was obtained.
16 The sample-containing cup was placed on the sample lift of the Lab station and raised into position using the Raise-Lower lever.
17 The trigger on the RemScan device was pressed and the TPH concentration displayed on the PDA after 15–20 s.
18 Each soil sample was scanned five times with thorough mixing between the scans and the TPH concentration was recorded after each scan. The average of the five scans was recorded as the final TPH concentration in (mg kg^−1^) of dry soil.

1 *Sample preparation*
2 Test soil samples were prepared as shown below:
3 Soil samples (n = 84) were collected from different bioremediation treatments.
4 Contaminated soil samples (n = 16) were collected from hydrocarbon-contaminated sites in Australia.
5 Soil samples (n = 5) were spiked in the lab with a relatively high concentration of diesel (≈100,000 mg kg^−1^)
6 Soil samples were collected from all treatments using a Simple Random Sampling technique [7], so the sample represents the entire treatment.
7 About 60 g of each soil sample was air-dried for 24 h at room temperature.
8 Large clumps of the dried soil were ground using a pestle and mortar, sieved using a 2 mm sieve and then mixed thoroughly until fully homogenized.
9 *RemScan set up*
10 The RemScan was installed on the Lab station and locked with the strap provided (Fig. 1.A). The Lab station is a steel scaffold structure that can hold the RemScan device on the top and has a sample lift located in the middle. The scaffold is connected to an air pump in order to move the sample left up and down using the Raise-Lower lever. The use of the Lab station helps to minimise human errors associated with instability of the operators’ hands.

## Method validation

The scanned soil samples were sent to Australian Laboratory Services Pty Ltd. (ALS), an accredited external laboratory. ALS uses the UPEPA 5030 and 8260 methods for measuring TPH in soils. TPH concentration was quantified against alkane standards C_10_–C_40_.

**Fig. 2:**
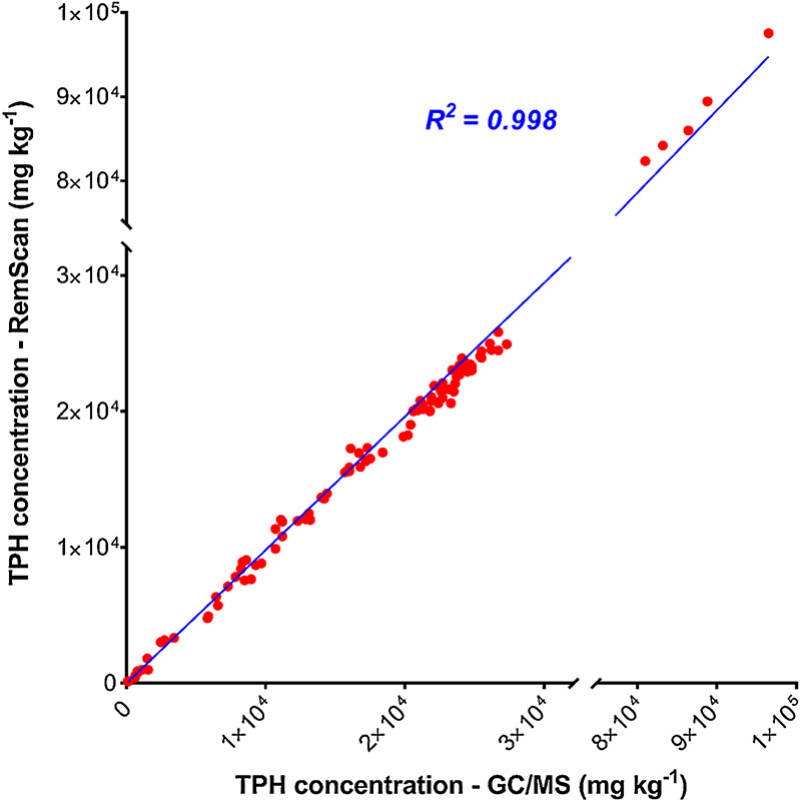
RemScan versus GC/MS values of TPHs concentrations (n = 105).

To determine the accuracy, the RemScan TPH values were scatter-plotted against the concentrations obtained from ALS, using Prism – Graphpad 7 software. The correlation coefficient (R^2^) showed that these two methods were 0.998 correlated in measuring the TPH concentration for the test soil samples (Fig. 2). Furthermore, a similar correlation was acquired using the Pearson correlation coefficient between the two groups of data. A comparison of the TPH concentration values measured using both methods for a subset of the test samples (*n = 20*) is shown in Table 1. Statistical analysis confirmed that no significant differences between the variances (F – test, α ≤ 0.05) were observed.

**Table 1:** TPH concentrations analysed using RemScan and GC/MS for a subset of the test soil samples (n = 20).

Sample ID	RemScan TPH values (mg/kg^−1^)	GC/MS TPH values (mg/kg^−1^)
HP 1	150	160
HP 2	560	660
HP 3	770	800
HP 4	850	860
HP 5	890	800
RB 1	8700	9280
BR 2	9920	10700
RB 3	11,380	10,700
BR 4	12,520	13,100
RB 5	15,620	16,000
BR 6	20,100	20,900
RB 7	21,880	22,100
BR 8	22,080	22,700
RB 9	22,900	23,900
BR 10	23,040	23,400
HC 1	82,340	81,000
HC 2	84,200	83,200
HC 3	86,000	86,400
HC 4	89,460	88,800
HC 5	97,560	96,500

The total cost of TPH analysis for the test samples by the external laboratory was around AU$ 40 per sample. In contrast, the cost associated with measuring TPH concentration using RemScan was AU$ 5 per sample.

Furthermore, the time required by the external laboratory for the TPH analysis results to be reported was about 5 working days for a dozen samples. The same number of samples needed only 2 h for the TPH concentrations to be measured (10 min/sample).

## Additional information

Petroleum hydrocarbons have become one of the most worldwide-spread contaminants as a result of global use, transportation and storage of oil [1]. Total Petroleum Hydrocarbons (TPH) are the major component of crude oil. TPH comprise a mixture of chemicals composed largely of carbon and hydrogen and consist of a major group of short and long-chain aliphatic hydrocarbons and a minor group of aromatic compounds. Exposure to TPH may cause permanent damage to the central nervous system; in addition, many compounds associated with petrogenic contamination are carcinogenic [2].

Bioremediation represents a simple, environmentally safe and cost-effective technique to remediate hydrocarbon-contaminated soil [3]. The bioremediation treatment, however, can represent a relatively long process (months to years) and requires regular monitoring of the TPH concentration to evaluate the efficacy of the treatment. Traditional laboratory techniques, such as Gas Chromatography/Mass Spectrometry (GC/MS) and Gas Chromatography/Flame Ionization Detector (GC/FID) are commonly used methods for measuring TPH concentration. However, these techniques are labour-intensive, expensive and time-consuming [4]. In addition, many of the solvents used for hydrocarbon extraction may pose both environmental and human risks [5].

Recently, a quantitative analysis of TPH in contaminated soils was performed using RemScan^™^, a new portable device marketed by Ziltek Pty Ltd. [6]. RemScan uses a diffuse reflectance (mid)-infrared Fourier transform (DRIFT) spectrometer and has the advantages of speed and low cost and yet represents an accurate way of estimating TPH concentration.
